# Dr. Baron's Pathological Opinions

**Published:** 1828-11

**Authors:** J. Baron


THE LONDON
### Medical and Physical Journal.

No 357, vol. lx.] NOVEMBER, 1828. [No 29, *New Series*.

For many fortunate discoveries in medicine, and for the detection of numerous errors, the world is indebted to the rapid circulation of Monthly Journals; and there never existed any work, to which the Faculty, in Europe and America, were under deeper obligations, than to the *Medical and Physical Journal of London*, now forming a long, but an invaluable series.—RUSH.

### ORIGINAL PAPERS,

AND

### CASES OBTAINED FROM PUBLIC INSTITUTIONS AND OTHER AUTHENTIC SOURCES.

### DR. BARON'S PATHOLOGICAL OPINIONS.

*To the Editors of the London Medical and Physical Journal.*

*Glocester; October 3d*, 1828.

Gentlemen,—In your Number for August, you were kind enough to insert a communication from me. On the 7th of that month, I thought it my duty to transmit to the Editors of the Edinburgh Medical and Surgical Journal the paper marked (A), together with a short note, requesting that it might be inserted in their next Number. On the 26th of the same month, the paper was returned to me, together with the note marked (B.) To that note I replied in the letter marked (C.)

As your goodness in printing my first paper has in some degree made you parties to these transactions, and as the Editors of the Edinburgh Journal have declined to comply with my just request, by observing a total silence, concerning the matters put to them in my letter (C), it seems but fair to lay all the documents before you: and, if you agree with me, you will lay them before the public.

I remain, gentlemen,

Your obliged and faithful servant,

J. BARON.

### (A.)

### *Observations on Changes of Structure in Organised Bodies.*

By John Baron, M.D. F.R.S. &c. &c.

I trust you will do me the favor to give a place to the following remarks in your next Journal. It appears to me that you have (unintentionally, I would hope,) given representations concerning some opinions of mine, which are certainly far from being correct. I have been too long engaged in the active and arduous practical duties of our profession to be very fond of hypothetical or conjectural reasonings; and I should think my time very much misspent were I to attempt to gain them any favor in the sight of my brethren. It is apparent, from Reviews of two of my works which have appeared in your Journal, that you do not consider me as having avoided errors of this kind. On the contrary, it is asserted that I have indulged in them in no common degree. It is my present purpose to point out to you some facts which have escaped your notice, and which may probably induce you to alter your opinion.

What I have now to say will apply chiefly to your Review of my "Delineations." Were I to remain silent, it might be supposed that I had assented to your representations, and the cause of useful knowledge might thereby be injured. If I understand the gist of your remarks, they lead to the following inferences: First, that none of my facts apply to pulmonary tubercles in man, and that I have taken for granted the thing to be proved;—secondly, that the whole of the doctrines which I have endeavoured to unfold, respecting the origin, progress, and character of a great variety of disorganizations, rest upon the same false foundation;—in short, that hypothesis, bare unsupported hypothesis, has been leading me astray for the greater part of my professional life. In corroboration of this representation, you affirm that I have no where stated that I have "traced the transformation from the vesicular or hydatiform condition to the opaque, firm, and tubercular structure in the tissue of the lungs; and it is only by applying to these organs what he (Dr. B.) recognises in the liver, that he ascribes to this source the formation of tubercles in the human lungs." (See vol. xxx. page 182.) You three times repeat this statement in the same article. As you cannot wish to maintain what does not accord with matter of fact, I am sure you will be glad to have an opportunity of correcting any inaccuracy into which you may have fallen. You will therefore do me the justice to attend to what follows.

From the very commencement of my inquiries, I have alluded to the difficulty of acquiring any thing like accurate knowledge of the primary or elementary condition of diseased structures in the human body; for this simple reason, that we seldom or never see them till all the original characters are lost. This is the cause that we have gained so little satisfactory information regarding the origin and course of morbid changes; that we are so little aware of the differences which exist between incipient and advanced disorganizations; and that we have hitherto been so unsuccessful in tracing the progress of alterations in structure. These convictions induced me to write as follows in page 21 of my "Illustrations:"—"When an individual affected with tubercles happens to be cut off by another disease, before the tuberculous affection has run its usual course, we may sometimes be presented in the same lung with examples of all the progressive changes which I have described. Such examples, of course, cannot often occur in the human subject. It has happened to me to meet with several of them, and I submit the following one to the reader's attentive consideration.

"A boy, about thirteen years of age, who had symptoms of pulmonary disease, was suddenly cut off by an affection of the head, and died on the 10th day of December, 1819. I examined the body on the following day. My principal attention was directed to the state of the thorax, and there I found most interesting illustrations of the description given above. There were accretions nearly of the whole of the right side of the chest, but they were not so firm by any means as they are in the more advanced stage of tuberculous disease. On examining the pleura, particularly towards its upper portion, it was studded with innumerable small bodies, many of them not so large as the head of a pin. They were perfectly transparent, and glistened on the surface of the membrane. On another portion of the pleura pulmonalis, I found a tubercle pendulous, as large as a pea, with thickened coats, and containing cheesy matter. This body is represented in plate 3d. *The transparent vesicles pervaded the substance of the lungs, as well as the membranes, but they did not all remain in this simple or elementary form. They exhibited every gradation in the progress which has been already described.* In their first state, neither lungs nor membrane, where they occurred, were much altered, but the condition of the surrounding lung became changed with that of the tubercles themselves. *Some had lost their transparency, and were of the size of millet-seed. Others were considerably larger, and were of a firm uniform consistence;*

*others were less uniform both in colour and texture.* Some had discharged their contents, and the empty cysts appeared: others, which were consolidated, had nearly coalesced, and formed a dense yellowish structure, quite foreign to that of the original pulmonary tissue." This statement was illustrated by two plates, Nos. 2 and 3.

From facts such as are recorded above, my description of the origin and progress of pulmonary tubercle was derived. Besides this positive testimony drawn from them, I have brought forward collateral proofs from the inferior animals. The glandered horse affords an example of genuine tuberculous disease of the lungs. My examinations of that disease prove that the progress is such as I have described. I have given one plate representing the incipient state of the disorder in the horse; and another which portrays corresponding changes in the lungs of the sheep. The description was drawn up with great care, and is, I believe, perfectly accurate. How, with all these facts before you, you could have asserted that I have no where traced the progress of the pulmonary tubercle, and that it is only by applying what I have recognised in the liver to a corresponding change of structure in the lungs, is to me inconceivable. You seem to declare that I have been guided by vague and unfounded analogy, and have allowed fallacious appearances to delude me throughout. I trust it will be found that, on the contrary, I have exercised a cautious and scrupulous discretion on this very question. I have in no instance inferred the progress of disease in one organ merely from what may be seen in any other. I have not rested any doctrine on comparative pathology alone; but I have availed myself of both these sources of information to elucidate and explain what, without such aid, must have remained obscure and unintelligible. When, therefore, I affirm that tuberculous disorganizations were common to every texture of the body, and that what was true of one organ was, *mutatis mutandis*, equally so of others, I was not influenced by analogical reasoning, but by positive and direct evidence. I had often, for instance, found these disorganizations in the membranes, in the viscera, and in other parts of the same subject. Examples of the same kind, without number, may be drawn from the writings of professional men. In aid of these facts there was the evidence deducible from the examination of diseased structure in the inferior animals. From the whole I arrived at this conclusion, that, though the symptoms and course of tuberculous diseases are exceedingly modified by the parts wherein they occur, their origin is regulated by general laws connected with the essential and fundamental properties of organised beings. The facts that support this opinion are clear, distinct, and to my mind conclusive. They embrace a great variety of the most interesting phenomena that pathology makes known to us. You have not given me any credit for fidelity in this matter, but would rather make it appear that *I* have arrived at conclusions without evidence, while it seems to me that *you* have neglected the facts I have adduced in the "Inquiry" and elsewhere, and have allowed preconceived notions to take place of solid observation.

What has just been said applies particularly to the objections which you have urged against my last publication. I have now to deliver a few remarks which have more direct reference to the general pathological doctrines at issue. I have urged them before, but unsuccessfully. Now I feel that they have still stronger claims to consideration.

I am very sorry that, with all my attempts to prevent misconception from the use of the word hydatid, I have not succeeded. I was fully aware of the evils that had arisen from mingling the zoological with the pathological question: and, although I did allude to the former in my first work, I took special care, then and on all subsequent occasions, to prevent error. It is, nevertheless, not unlikely that, in a subject which is admitted to be intricate and obscure, I may sometimes have failed.

My main object was, first, to ascertain the incipient or elementary state of various disorganizations; and then to trace their subsequent progress. As human pathology can only, in rare and uncertain instances, give us accurate intimation concerning these *principia morborum;* and as the *last* changes are exceedingly remote, in most of their characters, from the *first,* it was desirable to find out (if possible) some means of rendering this branch of knowledge more perfect and satisfactory than it ever can be whilst we trust to human dissection alone.

Fatal disorganizations in man seldom present to us an uniformity of appearance and texture. Some parts manifestly denote the greatest deviation from the natural state; while, on the other hand, we may detect portions where the departure from the healthy condition is very slight. This last point may be evinced in a still more striking manner when an individual is cut off by another disease soon after a disorganizing process has commenced. Another source of information is derived from witnessing the same disorganization in different stages, either in different parts of the same viscus or in different viscera of the same body. The result of observations of this kind goes to prove a connexion between things apparently dissimilar; to trace a progress where, at first sight, none could be detected; and to demonstrate that variations in appearance do not necessarily indicate difference of nature. All these points may be established by a reference to human pathology alone. That being done, we are in a condition to derive the greatest advantage from elucidating our imperfect information by facts and experiments drawn from the inferior animals. Analogies conducted in this cautious way will not mislead, and, if assiduously followed, are capable of imparting very valuable information. It has been my endeavour to keep these truths constantly in sight in the prosecution of my investigations. Till they are fully admitted by my professional brethren, I can scarcely hope that the facts which I have stated will gain the assent to which they are entitled.

I shall now add a few words to prove that, in the use which I have made of the term hydatid, I have neither acted inadvertently nor unadvisedly. SAUVAGES writes thus: "*Hydatides vero sunt* PRINCIPIA QUORUNDAM MORBORUM INTERNORUM, *sed hactenus signa desiderantur.*" MORGAGNI speaks of *hydatids degenerating into tubercles*, "as exemplified in the case of a virgin, in whom were various tubercles of different magnitudes, growing here and there to a sac in which a fluid had been contained, varying from the size of a large pea to that of the smallest hempseed, sometimes solitary, sometimes in clusters, but always scirrhous and hard; and, when cut asunder, discharging no fluid or gelatinous matter. Another instance which fell under his own immediate observation, as still more to the point, I give in his original words: "Et ne multis te detineam meæ me in albuginea et vaginali testiculorum tunicis persæpe habitæ observationes illuc adducunt ut credam hydatidum, *sive tunicarum in quibus increscunt ipsœ, membraenas laminas earum humorem complectentes, postquam disruptœ hunc effuderunt, se suaque vascula in carunculœ formam primum contrahere; et nisi novus illac humor effluere pergat, indurari et exsiccari denique sic ut alba illa et dura subrotunda* TUBERCULA *representant alia aliis, ut* HYDATIDES *fuerant majora aut minora*, &c. &c." (Vid. Epist. xxxviii. artic. 35.) What says BOERHAAVE on the same subject? "*Atque ita quidem harum nos rerum contemplatio ad hydatidas sensum speculatione hac deduxit. Qui sphœrici tumores liquida primo lympha targent, sensim degenerante, juxta varios in colore et crapitie mutata modos.*" (Vide H. Boerhaave Epist. Anat. ad Fred. Ruysch, p. 73.) Again, look at what DE HAEN

says in his Ratio Medendi, as well as in his Chapter de Hydrope cystico et Hydatibus, and you will find more than sufficient to justify me in the language that I have employed. I have referred to all these writers and in the " Inquiry" I quoted some of the passages at length. I then observed, that the origin of hydatids themselves was of less importance than the consideration of the vast " variety of formidable changes of structure to which they give birth." (See Inquiry, p. 111.) This observation was not written till after personal inspection had proved to me the accuracy of those distinguished authors whose names I have just mentioned. Were this a fit occasion, I could bring forward many additional proofs to corroborate what I have advanced. While relying on testimony of this kind, I little thought that it would be so much disregarded; and that it would be supposed that I was dealing in hypothetical and conjectural assertions, when I was in fact only elucidating the origin and cause of many disorganizations by clear and indisputable evidence. I then said, and I now repeat, that *that* evidence " illustrates the origin and progress of a great variety of the most fatal and alarming chronic diseases, which cannot be accounted for by any doctrines now in vogue, without involving the reasoner in the most palpable contradictions and inconsistencies." (See Inquiry, p. 117.)

I endeavoured, in a subsequent part of the same work, to give these facts a practical application, by pointing out the manner in which the actual appearances of different morbid growths are illustrated by a due consideration of the *principles* which I had before endeavoured to establish. These principles regard, first, the elementary condition of this genus of disorganization; secondly, the difference of appearance that may arise from the number, relative position, and progress of these elementary parts. Throughout the whole of this investigation, it was my object to state nothing that did not rest on unimpeachable evidence; and I have not yet discovered that the evidence is in any instance defective.

Divesting the subject of all the obscurity that might arise from the use of ambiguous terms, the sum of what I have said amounts to this,—that a great number of the most fatal disorganizations assume, at their commencement, definite and specific characters. The hydatid, as above explained, is unquestionably one of the most common of these forms. I am unable to conceive any chain of evidence more complete than that which bears upon this point. I would rest the proof, not upon any thing that I myself have seen, but upon the testimony of every accurate observer who has faithfully recorded what he has witnessed in his own dissections. If my professional brethren would only free their minds from preconceived opinions, and look at the subject simply, I feel quite assured that it would soon gain their assent. But, however this may be, it is fair to state that almost all that has been advanced regarding the progress of certain disorganizations that wear a complete aspect is capable of the most rigorous demonstration, whatever doubt may be entertained as to their origin.

It may still further simplify this subject to view it in another light. Let us put aside all technical terms, and consider dispassionately the following questions: First, are there any indications by which the primary deviations from healthy texture may be detected? Secondly, have these indications been seen by persons competent to judge of the subject? Thirdly, has the progress from the primary indications to the more advanced stages of disorganization been traced with care and accuracy? And, finally, what is the class of disorganizations to which testimony of this kind applies?

It cannot be doubted that these questions can only be answered in one way by all who will take the trouble to acquire the necessary information. They embrace the *principles* for which I have been contending. I took my ground on the basis of facts recorded by Sauvages, Morgagni, Boerhaave, Haller, De Haen, Turner, and many other high authorities, my own observations fully according with theirs. The facts alone I endeavoured to apply in explanation of many of the most common and fatal disorganizations: and, till such facts are overthrown, I shall deem the ground on which the pathological subject stands unshaken.

(B.)

*Edinburgh; August 21st,* 1828.

The Editors of the Edinburgh Medical and Surgical Journal have received the communication of Dr. Baron. They are at all times anxious to give gentlemen opportunity of correcting any misstatements of facts, or misrepresentations of arguments, which they may conceive have been given in accounts of works: and with this view they took some pains to compare the account of Dr. Baron's work given in the July Number with the original, and with the observations sent. They regret extremely that they cannot agree with Dr. Baron in regarding the account given in that Number as misrepresentation; and if, in the statement there exhibited, they decline to adopt the views of Dr. Baron, this is totally unconnected with any desire to misrepresent them, of which indeed they are entirely unconscious. For this reason, they cannot perceive that the insertion of the observations of Dr. Baron is either necessary or expedient.

Independent of this, however, they have made it an invariable rule, for reasons which must be obvious, never to insert papers which have already appeared in other Journals. As Dr. Baron must be aware that the paper sent to the Edinburgh Journal has appeared in the Medical and Physical for August, and therefore falls under this exception, he cannot, it is hoped, be offended that a rule which is absolute and imperative is not dispensed with in his case. The Editors of the Edinburgh Journal cannot conclude without assuring Dr. Baron that, though they cannot, in the present stage of the inquiry, adopt all his opinions regarding the formation of tubercles, they entertain a high admiration for the zeal and diligence with which he continues to cultivate the science of pathology.

*To Dr. Baron, Glocester.*

(C.)

*To the Editors of the Edinburgh Medical and Surgical Journal.*

*Glocester; August* 28, 1828.

Gentlemen,—I have received your note, together with the communication I forwarded to you for your next Journal. I confess I am somewhat surprised at your decision concerning that communication, and I feel myself constrained to address a few words to you on that subject. I have not said that you have wilfully misrepresented my statements. My words are, "that you have misapprehended my meaning, and (inadvertently, I would hope,) not accurately represented my sentiments." These words occur in the Medical and Physical Journal for August. The expressions I have made use of to yourselves are of like import. I say, "It appears to me that you have (unintentionally, I would hope,) given representations concerning some opinions of mine which are certainly far from being correct."

You tell me that you have compared the Review with my writings, and with the communication which I have sent, and that you can find nothing to justify such expressions: nothing to justify me in seeking to explain in your pages that which has been, I think, not accurately represented. Allow me to ask you, gentlemen, whether you are prepared to assert that the words which I have quoted from your Review of my "Delineations" are correct, and whether you are prepared to sustain their accuracy? Is it your intention, by the answer which you have sent to me, to affirm that I have no cause for complaint, and that it is inexpedient and unnecessary that my explanation should be attended to? I trust to your sense of justice to answer these questions distinctly, because, unless you are disposed to go thus far, it does appear to me that the other reasons you have assigned for not inserting my paper cannot be maintained. I have laid before you a distinct grievance, for which I sought redress; and I cannot conceive that I ought to be refused what is so just and reasonable, even though I did take the opportunity of stating part of my grievance in another Journal. My having done so seems to me to afford you the only plausible ground for your decision: but even this, on consideration, I think, will not avail you.

I am told by you that the paper which I sent had been previously published, and that it is an invariable rule with you not to give admittance to such articles into your Journal. It is true that the facts which expose the inaccuracy of your statements have been published before; but this, as I have already said, ought not to make against me in seeking redress at your hands. Some of the sentiments, too, are also in the Medical and Physical Journal; but the whole article is so different in arrangement and in language, that the one cannot be taken for the other. I have moreover to affirm, that there are in the article which I sent to you many important statements and quotations which are not in the other: so many, indeed, as to entitle me to consider it as a new article altogether, and therefore not coming within the rules that you have laid down.

Believe me, gentlemen, it affords me no satisfaction to gain assent to opinions because they happen to be mine; and did I not consider that truth and useful knowledge were concerned, I should not deem it necessary to trouble you or myself on this subject.

I am, gentlemen, your obedient humble servant,

J. BARON.

